# Genetic analysis of the Vitamin D receptor start codon polymorphism (FokI) in cervical vertebra and lumbar spine pathologies: a meta-analysis

**DOI:** 10.18632/oncotarget.20380

**Published:** 2017-08-21

**Authors:** Xinyu Hu, Min Liu, Yanjun Ni, Guolong Zhang

**Affiliations:** ^1^ Department of Scientific Research, Jiading District Central Hospital Affiliated Shanghai University of Medicine and Health Sciences, Shanghai, China; ^2^ The Former Dalian Sanatorium of Shenyang Military Region, Dalian, Liaoning, China

**Keywords:** Vitamin D receptor (VDR), FokI polymorphism, spinal diseases, cervical vertebra and lumbar spine pathologies

## Abstract

**Background:**

Vitamin D receptor (VDR) FokI polymorphism has been reported to influence the risk of spinal diseases. However, several studies suggest inconsistent results. Therefore, we performed this analysis to reveal the accurate relationship between VDR FokI polymorphism and spinal diseases.

**Materials and Methods:**

8 articles accord with the strict inclusion and exclusion criteria. 1116 cases and 1263 controls are entered into this analysis. The pooled odds ratios (ORs) and 95% confidence intervals (CI) are calculated to evaluate the association between VDR gene polymorphism and spinal diseases.

**Result:**

The results suggest that allele F is a risk factor for spinal diseases and the difference is significant (F vs. f: OR = 1.151, 95% CI, 1.020–1.300). For the genotype analysis of VDR FokI, no statistical differences exist in the models of heterozygote comparison (Ff vs. ff), homozygote comparison (FF vs. ff) and dominant model (FF + Ff vs. ff) (*p* > 0.05). However, in recessive model (FF vs. Ff + ff), there is a significant association between VDR polymorphism and spinal diseases (OR = 1.209, 95% CI, 1.017–1.436). In subgroup analysis, the results show that allele F is a risk factor for spinal diseases in each estimation. In hospital-based subgroup, the significant differences exist in FF vs. ff and FF vs. Ff + FF models. In degenerative spine diseases group, the results are consistent with that of overall studies.

**Conclusions:**

According to results of this meta-analysis, allele F is associated with the increased risk of spinal diseases. FF genotype may contribute to the susceptibility of spinal diseases. Therefore, VDR FokI polymorphism is related with spinal diseases.

## INTRODUCTION

Cervical spondylosis and lumbar spine pathologies are common diseases of musculoskeletal disorder. Neck and low back pain are the typical symptoms [1, 2]. The chronic hazards of these diseases are serious threat to human health. The previous researches show that compressive forces on vertebral endplates, obesity, bone mineral density and occupational factors are associated with the disease of vertebral body lesions, such as lumbar spine pathologies, lumbar disc herniation, degeneration of lumbar disc and spinal stenosis, cervical spondylotic myelopathy, ligament disease and so on [3–5]. While recent investigations suggest that genetic factors are also important causes of cervical vertebra and lumbar spine pathologies [6–8]. Vitamin D receptor (VDR) gene is the thoroughly researched candidate gene, which is connected with vertebral diseases. The VDR gene is located on chromosome 12q13.11 and is consisted of 11 exons [9]. There are numerous single nucleotide polymorphisms (SNPs) of VDR gene. FokI (rs2228570), BsmI (rs1544410), ApaI (rs7975232) and TaqI (rs731236) are the most frequently investigated SNPs of VDR gene [10]. The FokI polymorphism is a C/T single nucleotide transformation on chromosome 12, which is considered to be one of the most crucial genetic factors affecting the incidence of vertebral diseases. This change induces the transformation from ATG to ACG, resulting in the alternation of protein product [11]. Although the relationship between vitamin D receptor (VDR) gene and spine pathologies is studied systematically, the research results are inconsistent.

Therefore, it is necessary to update the research results of the association between VDR FokI polymorphism and vertebral diseases, which could provide further evidence to discover the real relationship between them. In this study, a meta-analysis is performed to assess the available articles.

## RESULTS

### Search strategy and characteristics of eligible articles

**Figure 1:**
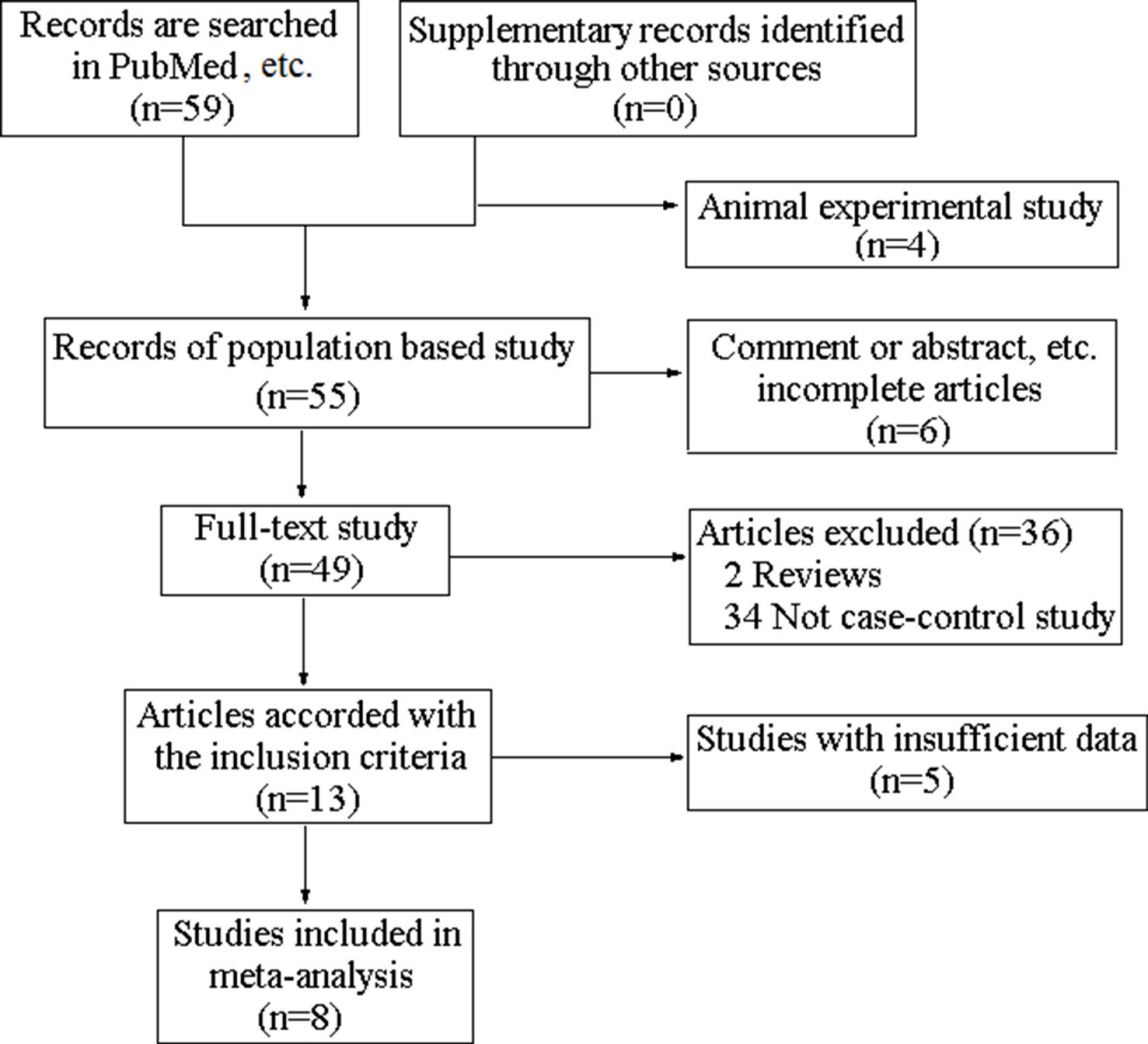
The flowchart of studies identification

The complete searching procedure is shown in Figure 1. 8 eligible studies including 7 hospital-based studies [17–23] and 1 population-based study [24] are collected in this meta-analysis on the basis of the inclusion criteria. The general information of the eligible articles including the first author, publication year, original country, disease category, control source, diagnostic method and genotyping technique are collected by two independent investigators. The characteristics of included studies are shown in Table 1. 1116 cases and 1263 controls from these articles are employed for the analysis of VDR FokI polymorphism. In addition, numbers of genotype FF, Ff and ff in two groups, the total case numbers and control numbers of included studies are collected to calculate the pooled odds ratio (OR) and the VDR FokI genotype distribution information are exhibited in Table 2.

**Table 1:** Characteristics of eligible articles

First author	Publication year	Country	Disease	Diagnostic method	Genotyping method	Control source	Score
Sansoni V	2016	Italy	Lumbar disc herniation	MRI	PCR-RFLP	HB	9
Colombini A	2015	Italy	Lumbar spine pathologies	MRI	PCR-RFLP	HB	8
Nikolova S	2015	Bulgaria	Idiopathic scoliosis	Radiodiagnosis	PCR-RFLP	HB	8
Vieira LA	2014	Brazil	Intervertebral disc degeneration	MRI	PCR-RFLP	HB	8
Colombini A	2014	Italy	Lumbar spine pathologies	MRI	PCR-RFLP	HB	8
Wang ZC	2010	China	Cervical spondylotic myelopathy	MRI	PCR-RFLP	HB	9
Kobashi G	2008	Japan	Ossification of the posterior longitudinal ligament	Radiography	PCR-RFLP	HB	9
Noponen -Hietala N	2003	Finland	Degenerative lumbar spinal stenosis	MRI	PCR-RFLP	PB	5

PCR-RFLP: polymerase chain reaction-restriction fragment length polymorphism

HB: hospital-based PB: population-based;

**Table 2:** VDR FokI genotype and allele distribution of eligible studies

First author	Case			Control			Case		Control		*P* value of HWE in control group
	FF	Ff	ff	FF	Ff	ff	F	f	F	f	
Sansoni V	53	44	13	44	51	15	150	70	139	81	0.971
Colombini A	117	120	30	101	117	36	354	180	319	189	0.821
Nikolova S	56	43	6	108	92	10	155	55	308	112	0.082
Vieira LA	17	50	54	10	46	75	84	158	66	196	0.434
Colombini A	117	120	30	89	99	32	354	180	277	163	0.601
Wang ZC	46	77	31	50	76	30	169	139	176	136	0.907
Kobashi G	31	21	11	37	70	19	83	43	144	108	0.132
Noponen-Hietala N	11	12	6	25	26	5	34	24	76	36	0.630

HWE: Hardy-Weinberg equilibrium

### Publication bias

**Figure 2:**
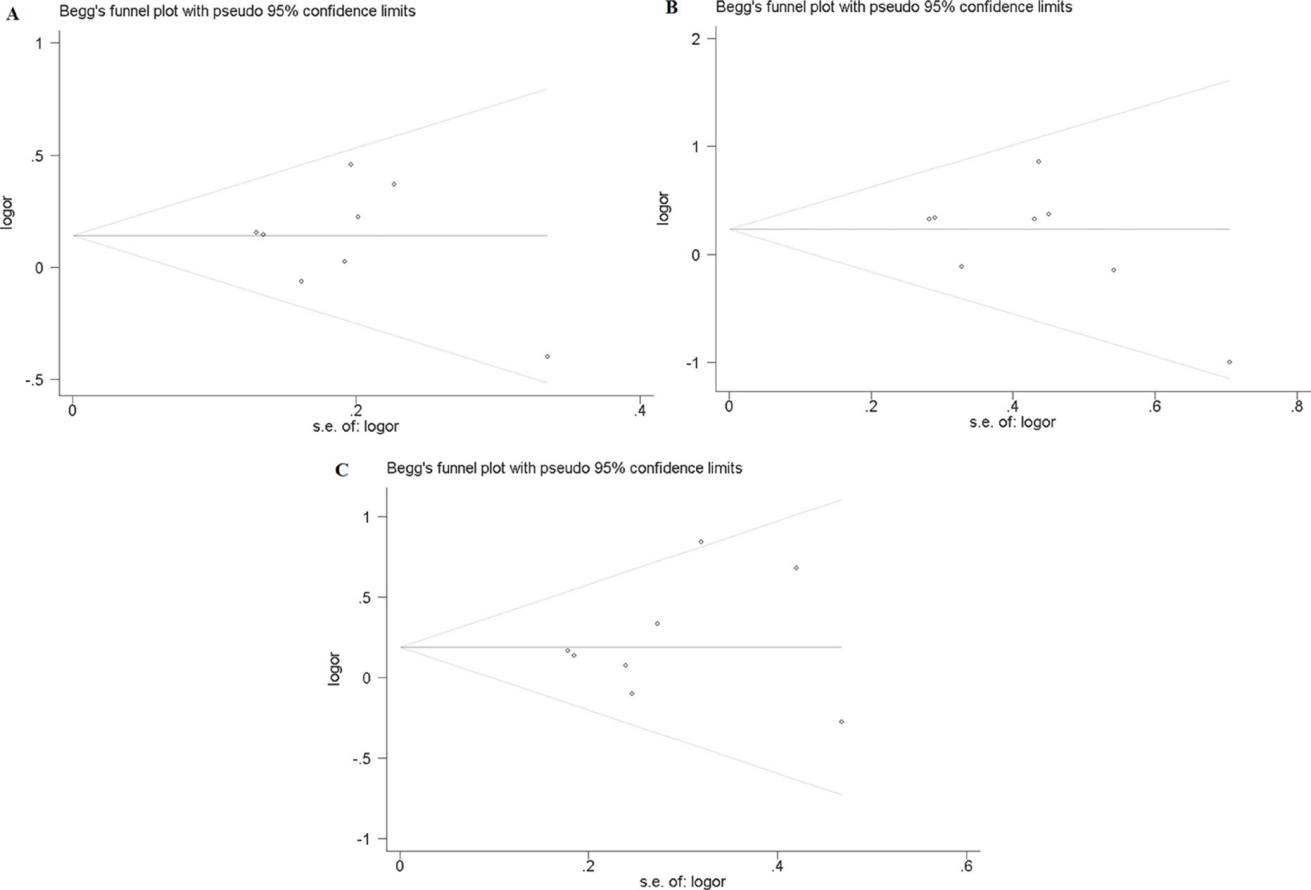
Publication bias of FokI polymorphism (**A**) Begg's test for publication bias in allelic model (F vs. f). (**B**) Begg's test for publication bias in homozygote comparison model (FF vs. ff). (**C**) Begg's test for publication bias in recessive model (FF vs. Ff + ff).

**Figure 3:**
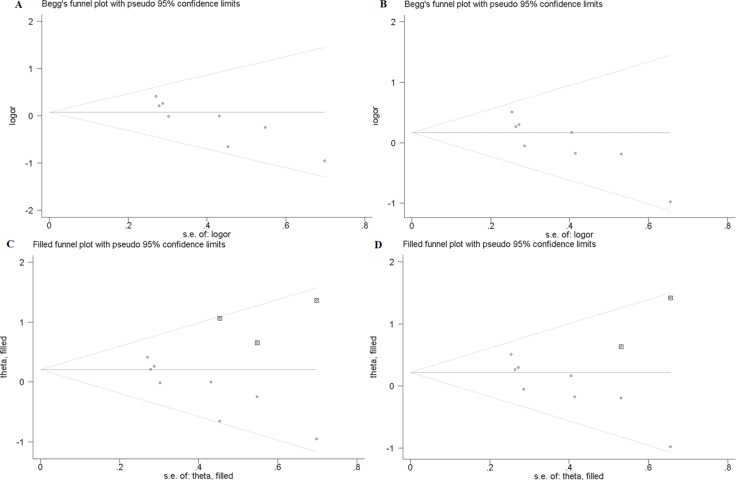
(**A**) Begg's test for publication bias in heterozygote model (Ff vs. ff). (**B**) Begg's test for publication bias in dominant model (FF + Ff vs. ff). (**C**) Trim and Fill for the bias analysis of Ff vs. ff. (**D**) Trim and Fill for the publication bias analysis of FF + Ff vs. ff.

In this study, publication bias is recognized by Begg's test. The results indicate that there is no notable evidence of publication bias in these comparison models, including the allelic model (F vs. f, Begg's test: *p* = 1.000), homozygote comparison model (FF vs. ff, Begg's test: *p* = 0.266) and recessive model (FF vs. Ff + ff, Begg's test: *p* = 0.902) (Figure 2A–2C). However, significant publication biases are detected in the model of heterozygote comparison (Ff vs. ff, Begg's test: *p* = 0.019) and dominant model (FF + Ff vs. ff, Begg's test: *p* = 0.019) (Figure 3A, 3B). “Trim and fill” method is performed to incorporate the hypothetical researches to recalculate the pooled risk assessment. After adjustment, the publication bias did not exist in the two models any more (Figure 3C, 3D). Therefore, there is no obvious publication bias in all comparison models.

### Meta-analysis

### Contrastive analysis of allele in all eligible studies

**Figure 4:**
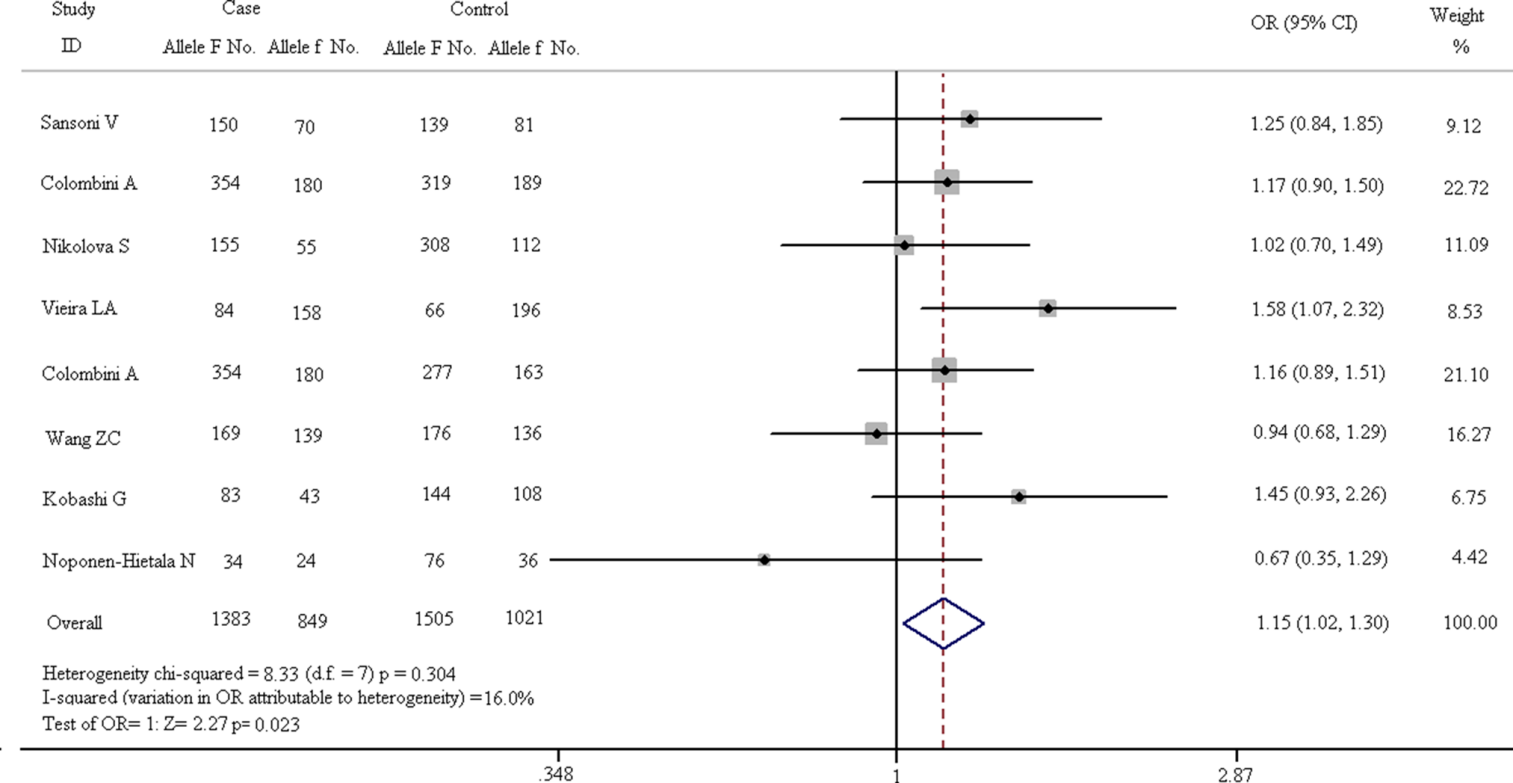
Forest plots of all eligible studies on allele of VDR FokI and the risk of spinal diseases

The results of VDR FokI allelic comparison (F vs. f) in case and control groups are listed in Figure 4. In fixed-effect model, the heterogeneity of subgroups have no statistical significance (chi-squared = 8.33, I^2^ = 16.0%, *p* = 0.304). The pooled results indicate that allele F is a risk factor for vertebral diseases compared with allele f (F vs. f: OR = 1.151, 95% CI, 1.020–1.300) and the difference is statistically significant (*Z* = 2.27, *p* = 0.023).

### Gene polymorphism analysis

**Figure 5:**
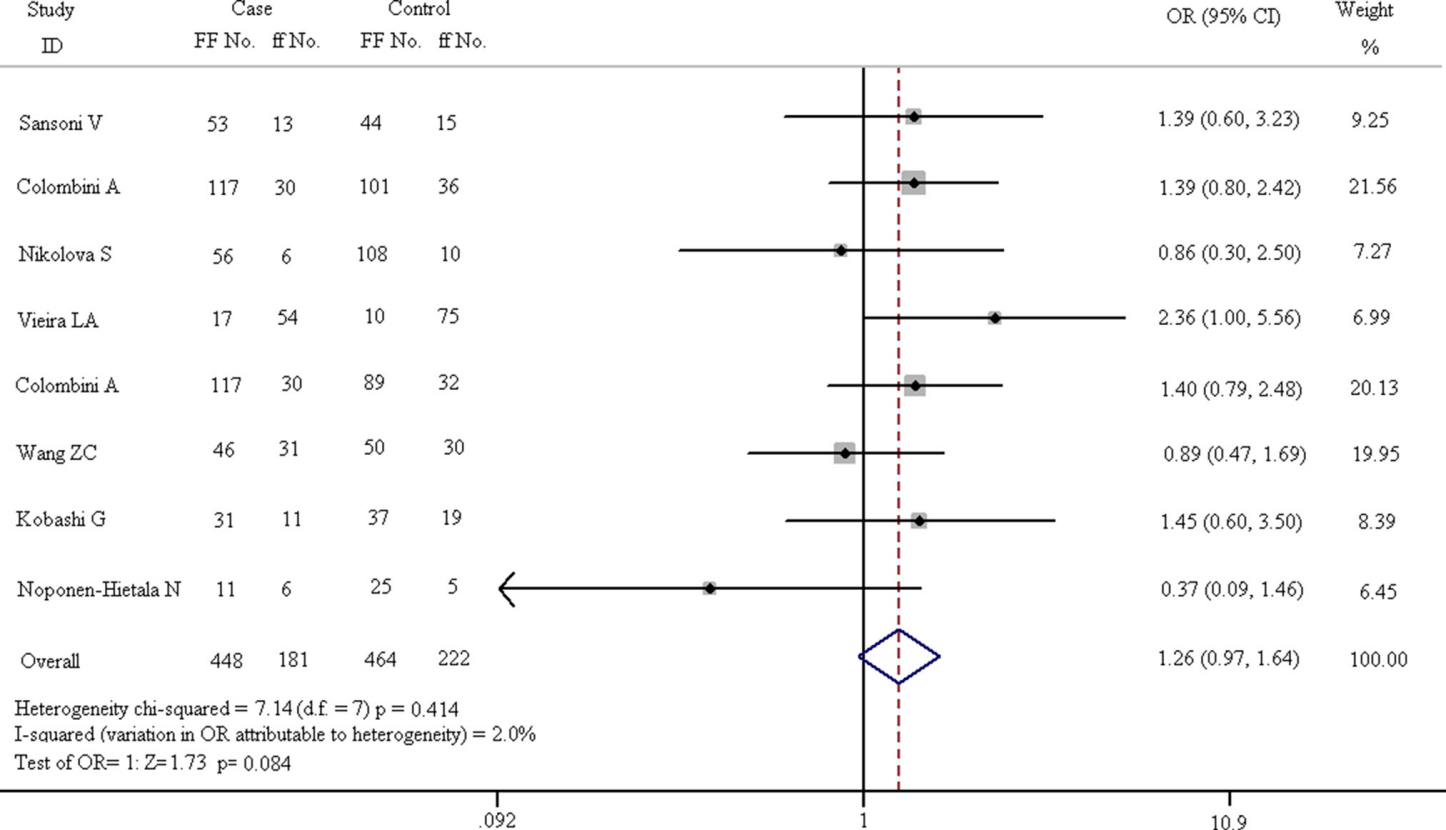
Forest plots of homozygote comparison (FF vs. ff) in all enrolled studies

**Figure 6:**
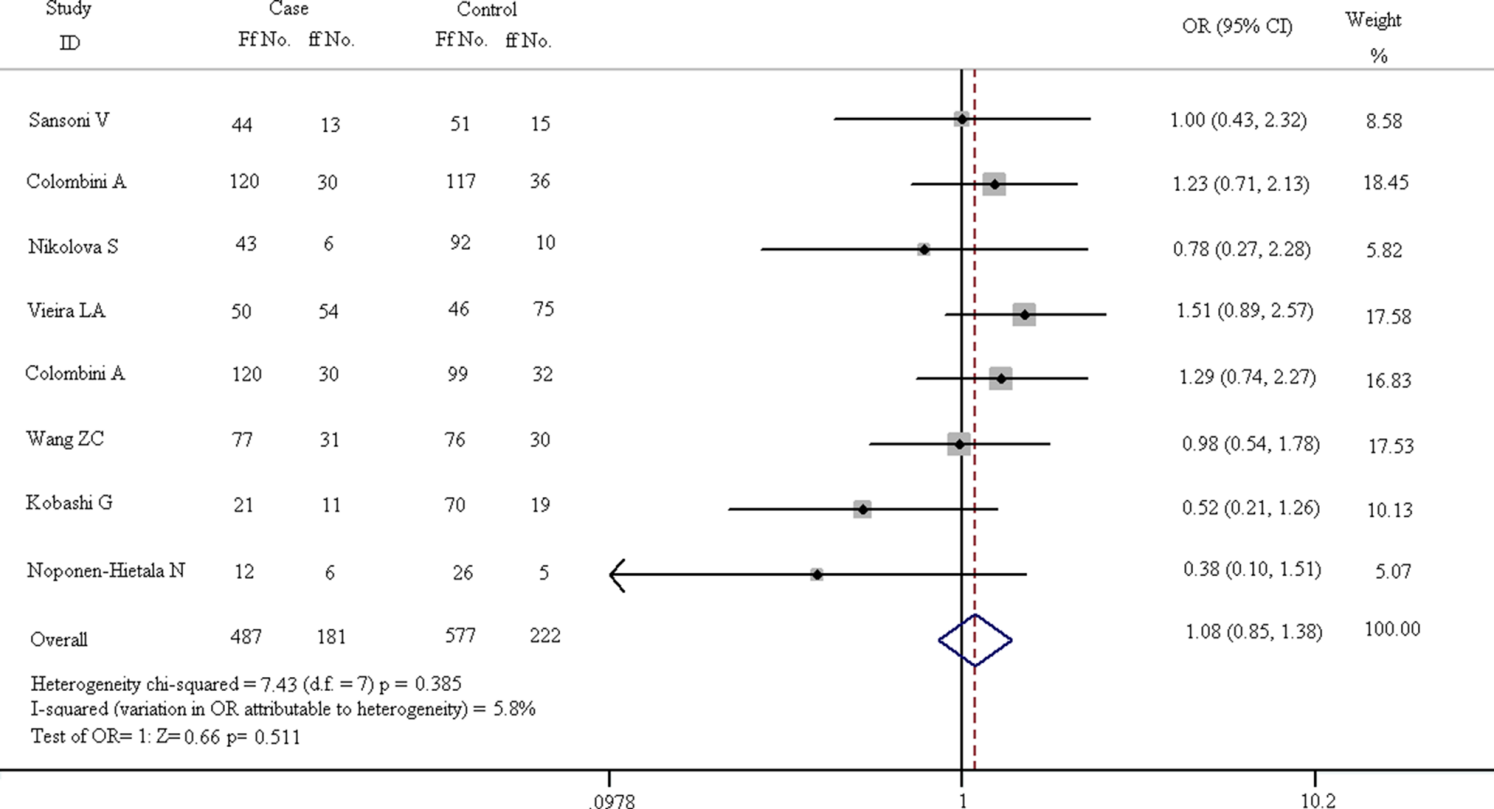
Forest plots of heterozygote comparison (Ff vs. ff) in all eligible articles

**Figure 7:**
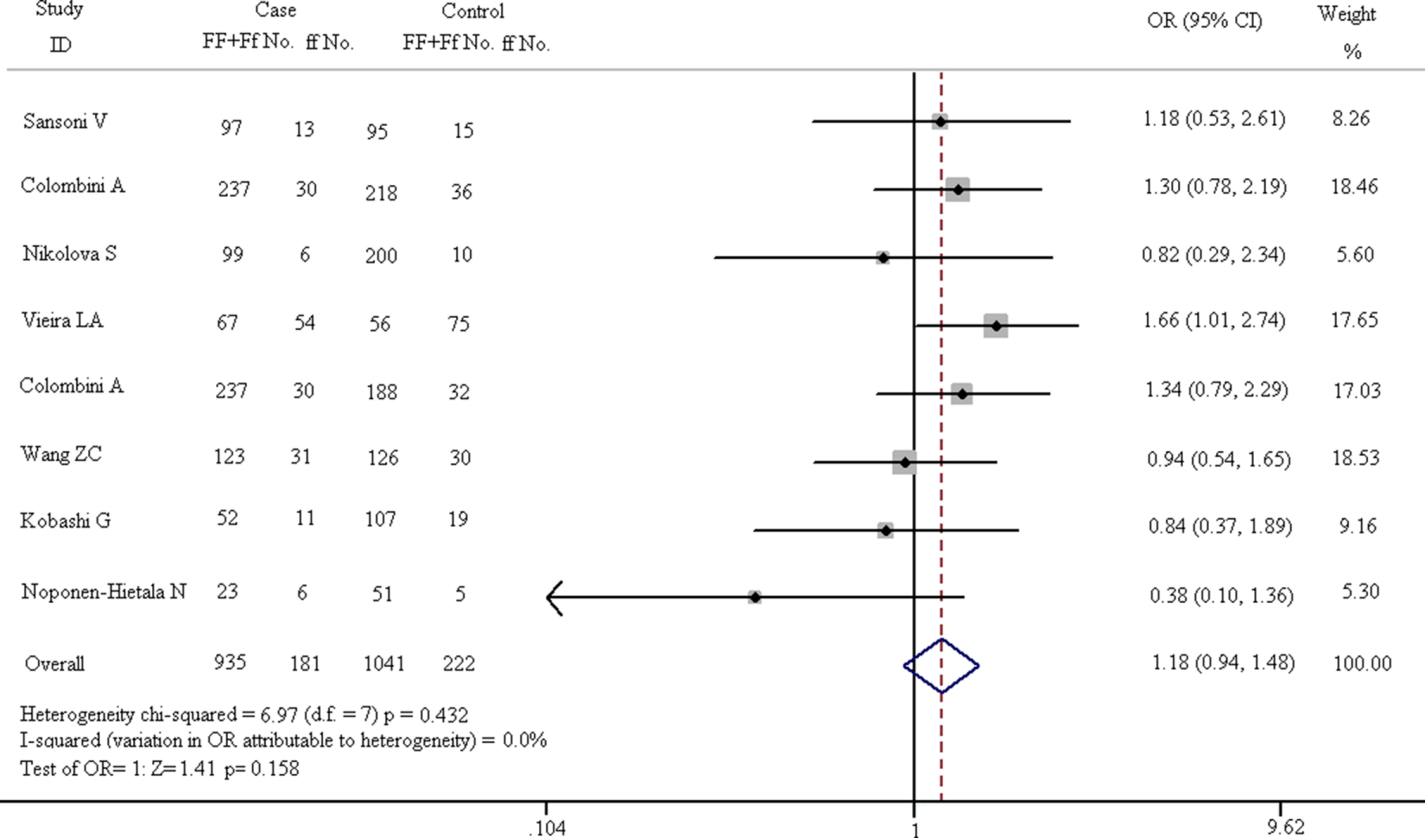
Forest plots in dominant model (FF + Ff vs. ff)

**Figure 8:**
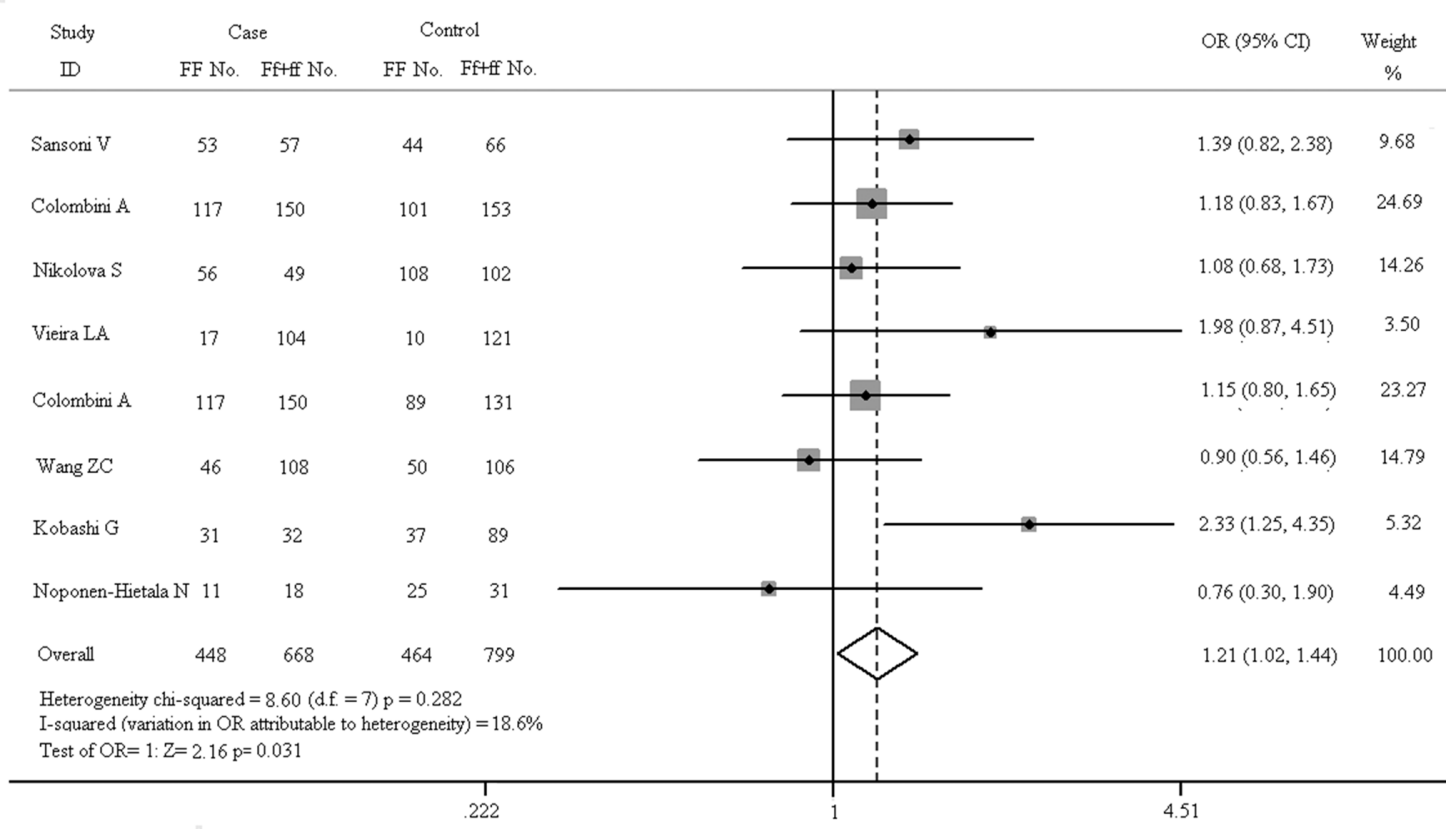
Forest plots in recessive model (FF vs. Ff + ff)

In order to reveal the relationship between FokI polymorphism and spinal diseases, subgroup analysis is divided into 4 parts: homozygote comparison (FF vs. ff), heterozygote comparison (Ff vs. ff), dominant model (FF + Ff vs. ff) and recessive model (FF vs. Ff + ff). The results of these subgroups are described in Figures 5–8. As shown in Figure 5, the results suggest that FF genotype is not a risk factor for spinal diseases. In fixed-effect model, the heterogeneity chi-squared value is 7.14 and there is no significant difference (I^2^ = 2.0%, *p* = 0.414). The pooled OR is 1.261 (95% CI, 0.969–1.641) and there is no significant difference in Z test (Z = 1.73, *p* = 0.084).

In Figure 6, it is shown that there is no difference in pathogenesis of spinal diseases between the Ff and ff carriers. The chi-squared test of heterogeneity indicate that there is no difference in subgroups on the basis of fixed-effect results (chi-squared = 7.43, I^2^ = 5.8%, *p* = 0.385). The pooled results of fixed-effect model suggest that Ff genotype is not a risk factor for spinal diseases compared with ff carriers (OR = 1.085, 95% CI, 0.851–1.382). The Z test of OR has no statistical differences (Z = 0.66, *p* = 0.511).

As shown in Figure 7, there is no obvious correlation between gene polymorphism and the increased risk of spinal diseases in the dominant model (FF + Ff vs. ff: OR = 1.179, 95% CI, 0.938–1.481). The results of Z test also present no significant association (Z = 1.41, *p* = 0.158). In fixed-effect model, the result of heterogeneity chi-squared test has no significant statistical differences (chi-squared = 6.97, I^2^ = 0.0%, *p* = 0.432). The results of recessive model (FF vs. Ff + ff) are shown in Figure 8. In fixed-effect model, the heterogeneity chi-squared value is 8.60 and there is no significant difference (I^2^ = 18.6%, *p* = 0.282). The pooled OR is 1.209 (95% CI, 1.017–1.436) and Z test shows significant difference (Z = 2.16, *p* = 0.031).

### Sensitivity analysis and subgroup analysis

### Sensitivity analysis

**Figure 9:**
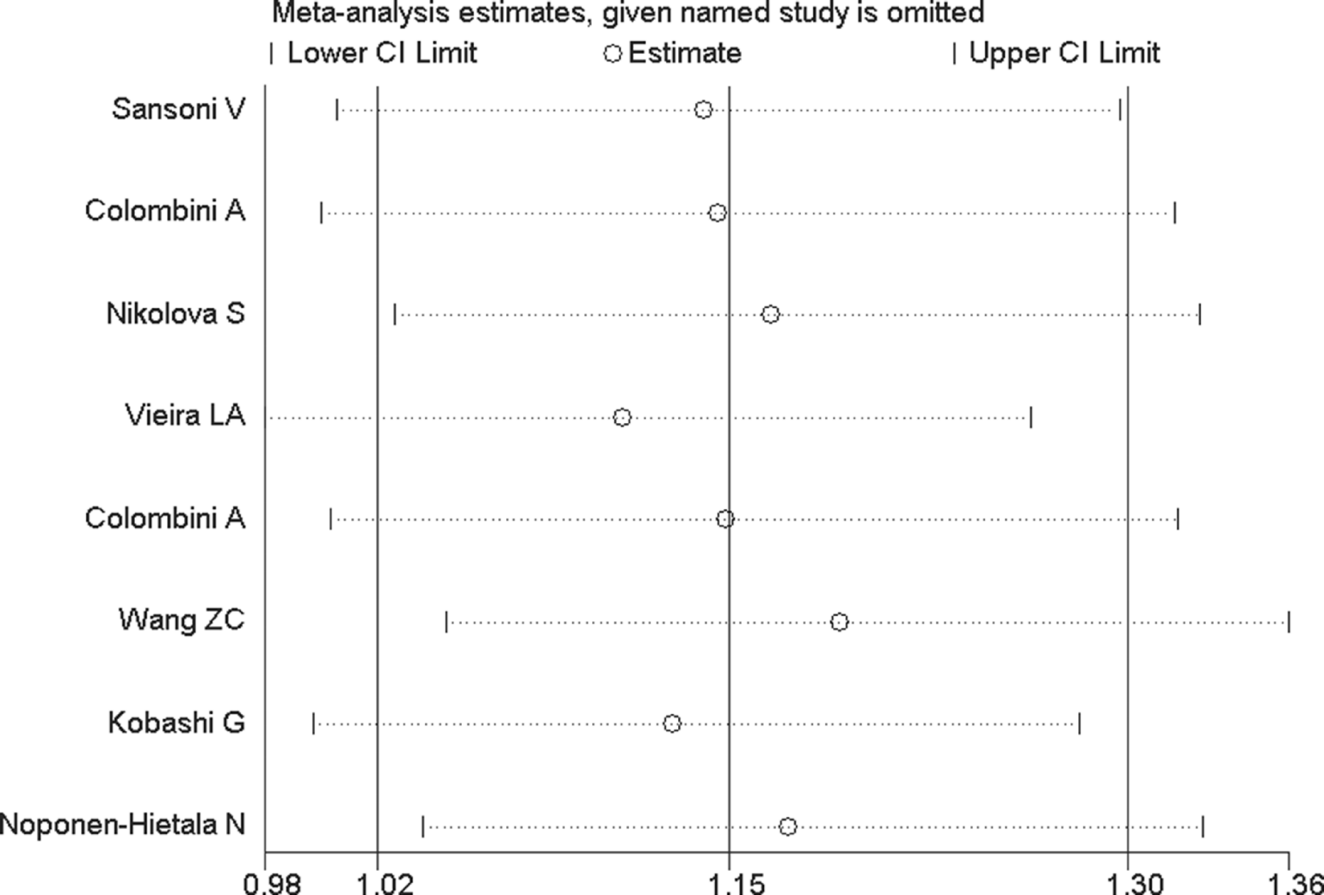
Sensitivity analysis of VDR FokI allelic comparison (F vs. f)

**Figure 10:**
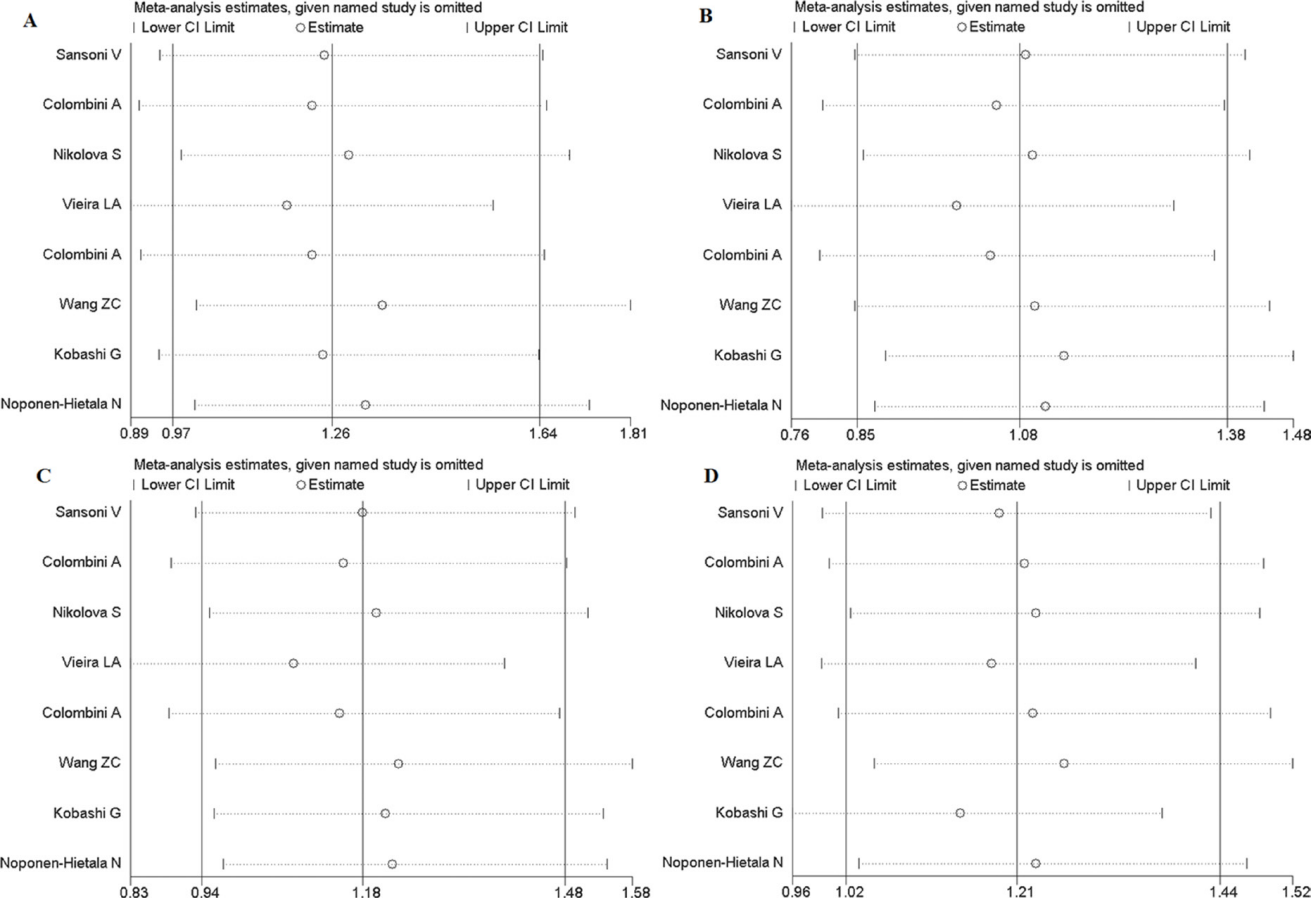
Sensitivity analysis of FokI polymorphism (**A**) Homozygote comparison (FF vs. ff). (**B)** Heterozygote comparison (Ff vs. ff). (**C**) Dominant model (FF + Ff vs. ff). (**D**), Recessive models (FF vs. Ff + ff).

In order to evaluate the influence of each study on the pooled OR, sensitivity analysis is performed and the STATA command “metaninf” is used. One study is expurgated from all eligible articles each time, then the new combined ORs are compared with the original pooled ORs, which are calculated using the data in all the eligible articles. As shown in Figure 9 and Figures 10A–10D, every removed individual article would not significantly affect the overall results, which suggests that the stability of this meta-analysis is high.

### Subgroup analysis

Source of control, quality of literature, population, diagnostic methods and classification of diseases are considered as influential factors for subgroup analysis. Noponen-Hietala N's study is population-based study, while, the quality of the evaluation results is low. Therefore, other 7 hospital-based studies have become a new research subgroup. Furthermore, subgroup analysis is also divided into caucasian group, MRI detection method group and degenerative spine diseases group according to ethnicity, different diagnostic method and classification of disease (Idiopathic scoliosis is excluded). The contrastive analysis results of these subgroups are shown in Table 3.

**Table 3:** Subgroup analysis of VDR FokI polymorphism on spinal diseases

Genetic model	Analysis model	Test of association		Test for heterogeneity	
		OR (95% CI)	*p* value	*I*^2^ (%)	*p* value
Hospital-based study					
F vs. f	Fixed-effect	1.174 (1.037, 1.328)	0.011*	0	0.464
FF vs. ff	Fixed-effect	1.323 (1.011, 1.731)	0.041*	0	0.682
Ff vs. ff	Fixed-effect	1.122 (0.877, 1.436)	0.36	0	0.522
FF + Ff vs. ff	Fixed-effect	1.224 (0.970, 1.554)	0.089	0	0.669
FF vs. Ff + ff	Fixed-effect	1.230 (1.032, 1.466)	0.021*	20.7	0.271
Caucasian population					
F vs. f	Fixed-effect	1.17 (1.019, 1.343)	0.026*	12	0.338
FF vs. ff	Fixed-effect	1.343 (0.989, 1.824)	0.059	13.2	0.330
Ff vs. ff	Fixed-effect	1.189 (0.900, 1.572)	0.223	0	0.514
FF + Ff vs. ff	Fixed-effect	1.282 (0.985, 1.667)	0.065	5.9	0.379
FF vs. Ff + ff	Fixed-effect	1.190 (0.981, 1.445)	0.077	0	0.710
Diagnostic method (MRI)					
F vs. f	Fixed-effect	1.144 (1.000,1.308)	0.049*	27.9	0.225
FF vs. ff	Fixed-effect	1.277 (0.960,1.699)	0.093	23.7	0.256
Ff vs. ff	Fixed-effect	1.174 (0.906, 1.522)	0.225	0	0.542
FF + Ff vs. ff	Fixed-effect	1.238 (0.970, 1.581)	0.086	12.1	0.338
FF vs. Ff + ff	Fixed-effect	1.157 (0.953,1.405)	0.140	0	0.557
Degenerative spine diseases					
F vs. f	Fixed-effect	1.167 (1.026,1.327)	0.018*	24.3	0.244
FF vs. ff	Fixed-effect	1.292 (0.985,1.696)	0.064	9.4	0.357
Ff vs. ff	Fixed-effect	1.104 (0.861,1.415)	0.437	15.0	0.316
FF + Ff vs. ff	Fixed-effect	1.20 (0.949,1.516)	0.127	7.6	0.370
FF vs. Ff + ff	Fixed-effect	1.23 (1.022,1.480)	0.028*	28.1	0.214

* ***p*** < 0.05

## DISCUSSION

The onset of spinal diseases is affected by many factors. Behavioral factors, environmental factors and genetic factors are the most thoroughly researched influential factors. Currently, genetic factors turn into research highlights in spinal disease instead of non-genetic factors. A large number of studies indicated that VDR gene plays crucial roles in the etiology of spinal diseases. FokI, BsmI, ApaI and TaqI are the most common types of VDR gene. Various studies have shown that FokI polymorphism is the only one that results in different structure of VDR protein and it is associated with increased risk of spinal diseases [25]. However, other investigations suggest that the FokI polymorphism of VDR is unrelated with the pathogenesis of spinal diseases. The conclusions of these studies are inconsistent. Therefore, we conduct this meta-analysis to reveal the relationship between FokI polymorphism and spinal diseases. In the 8 enrolled studies, the results of 4 articles indicate that allele F and FF genotype of VDR FokI increased the risk of spinal diseases. Meanwhile, the consequences of other 4 studies are opposite. The results of all eligible articles in this meta-analysis illustrate that allele F is a risk factor for spinal disease with the comparison to allele f and the difference is significant difference (*p* < 0.05). In the recessive model (FF vs. Ff + ff), the results suggest that FF carriers have a higher risk of spinal diseases compared with the population who carry the recessive gene with statistically significant differences (*p* < 0.05). However, in the genotype analysis of VDR FokI including dominant and (FF + Ff vs. ff), heterozygote comparison (Ff vs. ff) and homozygote comparison (FF vs. ff), any kind of genotype do not increase the risk of spinal diseases and no statistical difference exists in any estimates (*p* > 0.05). In the eligible literatures, there is one literature with poor quality or different control source. The vast majority of eligible articles are hospital-based study and only one paper is population-based study. When the individual study is removed, the results of studies with hospital-based controls are slightly different from the results of all enrolled studies. We found that FF genotype carriers are vulnerably threatened by the spinal diseases compared with ff genotype and the population carried the recessive gene, meanwhile, the difference has the statistical significance (*p* < 0.05). Furthermore, subgroup analysis is also divided in to caucasian group, MRI detection method group and degenerative spine diseases group according to ethnicity, different diagnostic method and classification of disease. In the subgroup of caucasian and MRI detection method, only the difference in the allelic model (F vs. f) is statistically significant (*p* < 0.05). In degenerative spine diseases group, the results are consistent with that of all enrolled studies.

Our meta-analysis is quite rigorous and has many prominent advantages. First of all, this paper is focused on VDR FokI polymorphism and the risk of spinal diseases. Literatures are chosen from PubMed and Web of science etc., which are the open classic biomedical databases. A reasonable search strategy is designed. Language type and the period covered by the publications are also limited strictly. Secondly, reasonable search strategy, objective quality evaluation, specific inclusion criteria and strict exclusion criteria are conducted to ensure the credibility of this meta-analysis. Finally, suitable statistical methods are used to calculate the results. Sensitivity analysis and stratification analysis are also performed to control the confounding factors.

However, some limitations of this study should not be neglected. The results of the meta-analysis may be influenced by certain limitations. Firstly, pathogenesis of spinal diseases could be affected by non-genetic or genetic factors separately, that is certified by numerous investigations. However, it is worthy to notice that the interaction between genetic factors and non-genetic factors may impact the occurrence of spinal diseases. For example, genetic factors with the interaction of environmental factors increase the risk of spinal diseases. Secondly, confounding variables such as sex, age, smoking and alcohol habits, etc. should be considered accurately and controlled preferably. The data of this meta-analysis are collected from published literatures and it is impossible to eliminate publication bias completely. We can only minimize the effect of publication bias to obtain more reliable results.

## MATERIALS AND METHODS

A detailed literature search strategy is performed using the keywords “Vitamin D receptor (VDR)”, “FokI polymorphism”, “cervical vertebra pathologies” and “lumbar spine pathologies” to search all the related articles in PubMed, which is a widely used online biomedical database (ultimate search updated on December 13, 2016). We also searched the papers in the frequently-used databases, such as “Web of Science”, “Medline”, etc. The search strategy consist of the terms “Vitamin D receptor (VDR)”, “FokI polymorphism” combined with “cervical vertebra pathologies” and/or “lumbar spine pathologies”. Two independent investigators screened the relevant articles using standardized screening guide.

### Inclusion criteria

a. The included studies must be concentrated on the relationship between VDR FokI polymorphism and the diseases of spine.

b. Case-control study is the unique methodology in selected articles.

c. Studies with enough data to calculate odds ratios and corresponding 95% confidence intervals (ORs, 95% CIs) are included.

d. Cases are diagnosed by diagnostic radiology and clinical diagnosis.

### Exclusion criteria

a. Abstracts, letters, comments, editorials, reviews, single-case reports and family-based studies are excluded.

b. The patients who received chemotherapy, radiotherapy or surgical operation treatment are excluded.

c. The articles with insufficient data or overlapped data are excluded.

### Quality assessment

We evaluate the quality of eligible studies in accordance with an improved 10-point scale, which is the appropriate quality assessment for case-control study [12, 13]. Quality evaluation parameters and standards of this modified scoring system (range, 0–10 points) is shown in Table 4, the higher score means the better quality of article. The average score for the eligible studies is 8 points.

**Table 4:** Quality evaluation parameters and criterion

Parameter	Score		
	2	1	0
Sample size	> 100	50–100	< 50
Control source	Hospital-based study	Population-based study	Unclear
Matching factors of case and control	> 3 factors	1–3 factors	Unclear
Basic information	Adequate	Sectional	Inadequate
Diagnostic method and genotyping method	Positive radiological diagnosis and PCR-RFLP	Positive radiological diagnosis or PCR-RFLP	Not commonly used method

PCR-RFLP: polymerase chain reaction-restriction fragment length polymorphism

### Statistical analysis

This meta-analysis is performed using STATA software (version 12.0, STATA Corp., College Station, TX, USA). Crude Odds ratios (ORs) and corresponding 95% confidence intervals (CIs) are calculated to assess the strength of association between VDR gene polymorphisms and the susceptibility of spinal diseases. Pooled ORs are calculated using the data of eligible articles in random-effect model (M-H heterogeneity method) or fixed-effect model (Mantel and Haenszel method). I^2^ index and *p* value of the chi-squared test are used to inspect the heterogeneity among enrolled literatures. If notable heterogeneity exist (*p* < 0.05 and/or I^2^ > 50%), the random-effect model is used to estimate ORs [14, 15]; conversely, the fixed-effect model is performed [16]. ORs are calculated in allele contrastive analysis (F vs. f), homozygote comparison (FF vs. ff), heterozygote comparison (Ff vs. ff), dominant (FF + Ff vs. ff) and recessive model (FF vs. Ff + ff). The Z test and *p* value of 0.05 are used to judge whether the differences of OR values have statistical significance. Stratification analysis is decided according to source of controls, ethnicity, different diagnostic method and classification of disease. The chi-square test for Hardy-Weinberg equilibrium (HWE) is performed and the *p* value greater than 0.05 is considered to be balanced in control population. Sensitivity analysis is conducted to assess the influence of individual studies. Begg's test is applied to evaluate the publication bias.
